# The importance of genome sequence quality to microbial comparative genomics

**DOI:** 10.1186/s12864-019-6014-5

**Published:** 2019-08-20

**Authors:** Theo H. M. Smits

**Affiliations:** Environmental Genomics and Systems Biology Research Group, Institute of Natural Resource Sciences (IUNR), Zurich University of Applied Sciences ZHAW, Wädenswil, Switzerland

**Keywords:** Assembly, Taxonomy, Genomes, Sequencing, Annotations

## Abstract

The quality of microbial genome sequences has been a concern ever since the emergence of genome sequencing. The quality of the genome assemblies is dependent on the sequencing technology used and the aims for which the sequence was generated. Novel sequencing and bioinformatics technologies are not intrinsically better than the older technologies, although they are generally more efficient. In this correspondence, the importance for comparative genomics of additional manual assembly efforts over autoassembly and careful annotation is emphasized.

## Main article

In my recent research, I have on several occasions dealt with bacterial genome sequences that were of low quality (here defined as “genome sequence assemblies that contain many contigs, and eventually with obvious misassemblies and unresolved plasmid sequences). A major problem is that the quality of these genome sequences is not indicated in the relevant databanks or in the associated literature, even though basic methods for genome quality assessment are available [1–3]. As some of the low-quality genomes can be of potential interest, we may invest considerable time to finally conclude that these genomes are not of much use for us. It is my opinion that this loss of time can be avoided by simple means.

New technologies are always taken skeptically. Already when I was working with 454 sequencing technology, homopolymers were a major concern [4]. The same problem was observed later with reads from IonTorrent systems [5, 6]. Assembly of short reads from technologies such as Illumina often yielded assemblies with a large number of contigs. Genome assemblies with long reads from PacBio SMRT sequencing or more recently Oxford NanoPore MinION sequencing are often superior in assembly due to the low number of resulting contigs (often complete bacterial genomes) but there are still concerns regarding the high error frequencies and reliability [7–9]. Many of these problems can be resolved by some time with an assembly specialist, improving the assembly quality remarkably.

The large number of contigs after assembly is one of the major problems that were observed when using short-read sequencing technologies. A recent publication on the intraspecies taxonomy of the plant pathogen *Pseudomonas syringae* included genomes with up to 5099 contigs [10]. The quality of these genome sequences may be fine for taxonomical analysis where most parameters like average nucleotide identities (ANI) [11] or genome-to-genome distance calculation (GGDC) [12] are not dependent on the integrity of annotations. However, for comparative genomics searching for individual gene sequences, these fragmented genomes are not applicable. Just do the back-of-the-envelope calculation: having a mean genome size of around 6 Mb per genome [10], this would indicate that the size of an average contig in a genome sequence with 5000 contigs would be around 1.2 kb. Having an average coding density of 85% and an average gene size of 1 kb for bacteria, this would indicate that there is maximally one full gene per contig, but it more often happens that you find two fragmented genes on the contig boundaries. This certainly limits the use of such an assembly.

It should be stated that often a large number of contig gaps cannot be resolved, but this is dependent on the genome. We recently sequenced two genomes of *P. syringae* using 2 × 300 base paired-end Illumina sequencing, and obtained a large number of contigs (214 and 246 contigs, respectively) [13]. In these genomes, many of the contig breaks are caused by the presence of insertion sequence (IS) elements. As IS elements are typically around 1.2–1.5 kb, a shotgun library with 500 bp inserts is not suitable for positioning the IS elements, present in multiple copies in the same genome. For this reason, our research group now prefers to use PacBio sequencing with a high coverage to improve the quality of genome assemblies from species that harbor a large number of IS elements [14, 15]. Still, manual inspection after sequencing was required to solve some sequence problems.

**Fig. 1:**
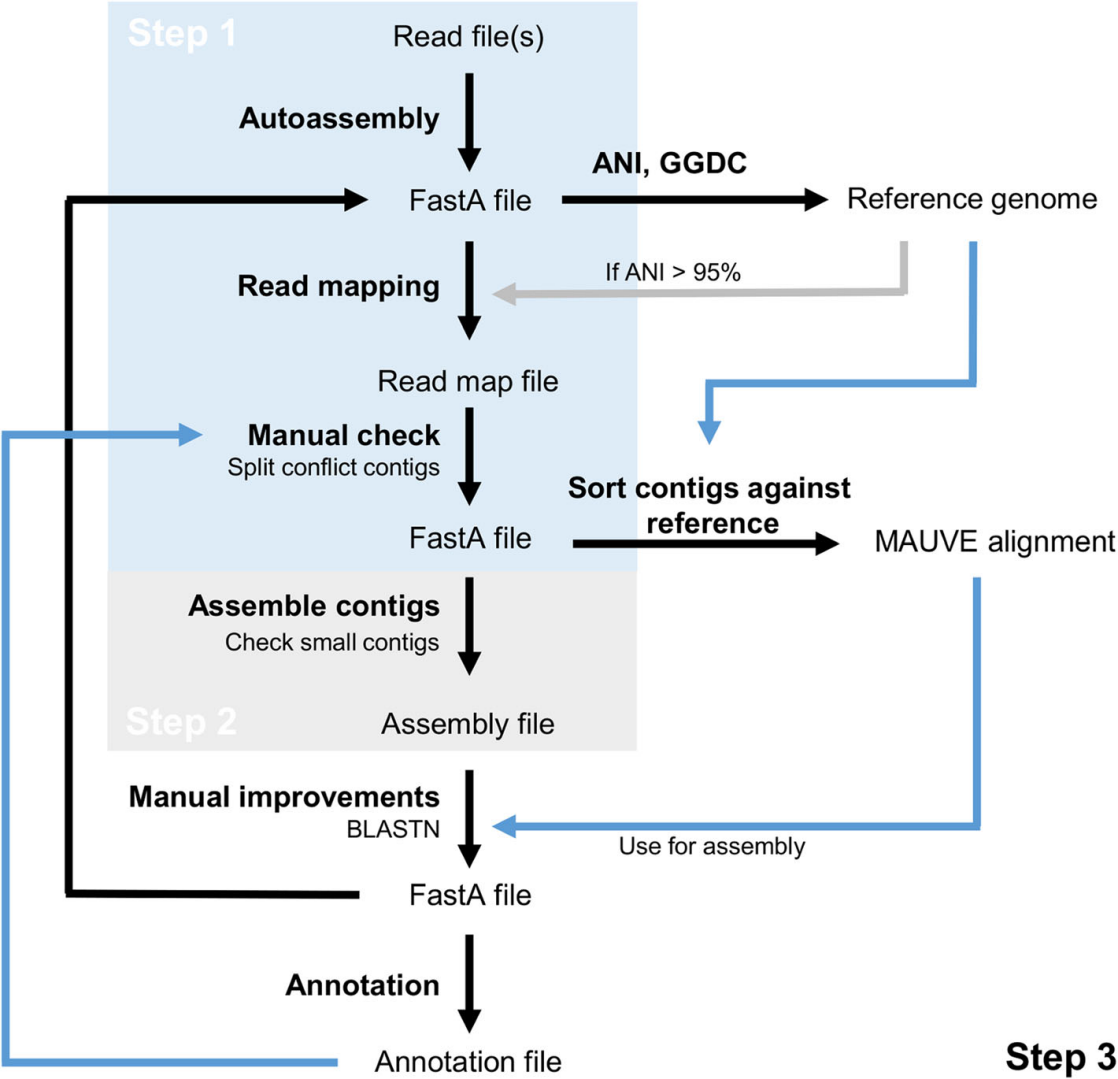
Flow diagram for high quality genome assemblies as used in the author’s institution. To follow the process described in the text, the parts involved in step 1 and step 2 are shaded, whereas all other processes belong to step 3. Black arrows: follow-up processes, blue arrows: information flow, grey arrow: potential follow-up process

On the other hand, it should also be stated that most genomes sequenced with Illumina technology can easily be improved in their quality by some additional steps of assembly (Fig. 1). Within our research group, we commonly spend up to one week per genome to reduce the number of contigs from an Illumina assembly. After autoassembly, we first perform a read mapping against the FastA file of the de novo assembly using SeqMan NGen (DNASTAR, Madison, WI, USA). This program has a special workflow, which allows the mapping of reads over the border of the contigs, which, when using 2 × 300 base reads, often gives more than 200 bp additionally on the left and right side of the contig. Manually checking the mapped reads in SeqMan Pro (DNASTAR) will uncover assembly errors based on false joints as these repeats will have a higher coverage on part of contigs than the average coverage. Such contig may be split before the next step.

The second step is to perform an assembly of all contigs from the resulting FastA file in SeqMan against each other. Here, several contigs may already be joined based on the additional sequence information, as overlaps are generated. Additionally, this process will eliminate many of the small contigs, which may be included inside other contigs. These will be checked if validly included. When a reference genome of the same species is available, this sequence can also be used to map reads against, followed by combining mapped and de novo contigs in SeqMan. However, this may introduce other problems due to misassembled regions.

Afterwards, the overlaps need to be checked carefully, as in case of contig forks, contigs may be joined erroneously. Read mapping using SeqMan NGen followed by manual analysis of mapped reads using SeqMan Pro can solve this kind of issues. When a complete genome, closely enough related as determined by ANI [11] or GGDC [12], is available, the program MAUVE [16] can be used to sort all contigs against the reference genome [17]. Using the synteny between the genomes from BLASTN analyses, several gaps may be closed. Others, potentially erroneously joined in the previous step, may have to be split again. The process has to be repeated several times to yield the FastA file of a final high quality draft genome assembly, as not all gaps can be resolved (e.g. rRNA operons). After annotation, information can be derived from the contigs that could lead to improved contig assembly, e.g., when a contig represents a plasmid.

The above mentioned process often yields closure of plasmid sequences from draft genomes [18], but also routinely a reduction of the total number of contigs to under 50 contigs per genome [19–21] with near complete removal of small contigs. Due to a thorough quality check at every assembly step by repeated read mapping and visual checking (Fig. 1), we make sure not to aggressively reduce the number of contigs by combining contigs that do not belong together [22, 23]. As the raw reads are generally available from databanks, the workflow (Fig. 1) would be possible for submitted genome sequences as well [24], but the effort is substantial and success is not guaranteed.

The problem with long-read technologies is not the number of contigs, but the quality of the individual read sequences. By using sufficiently large number of reads or additional reads from a short-read technology for assembly, the quality of the assembly can be improved significantly. However, if a genome is only used for. Taxonomic analysis, sequence errors based on lower coverage are not intrinsically detected. Unfortunately, such genomes will all the same appear in comparative studies, influencing their quality [25]. We recently retrieved the genome sequence, generated with MinION sequencing, of a bacterium described as “*Kluyvera intestini”* GT-16 [26]. This genome clustered closely to the genomes of two recently described novel species in the genus *Phytobacter* [27]. A simple test with ANI showed that strain GT-16 belongs to the species *Phytobacter diazotrophicus* (T.H.M. Smits and F. Rezzonico, unpublished). After the analysis of the genome sequence with the comparative genomics program EDGAR [28, 29] together with several other genomes of *Phytobacter* and related genera, we noticed that inclusion of the GT-16 genome sequence led to a drastic drop in the number of core genes. Reannotation using Prokka [30] did not improve the situation, and the summary of the annotation indicated a large number of pseudogenes. An examination of the annotation showed that these pseudogenes were caused from frame shifts, presumably originating in sequencing errors in the reads used. Interestingly enough, the same authors had previously published a draft genome of the same strain based on Illumina reads [31]. Combination of the data in a hybrid assembly approach would have yielded a high-quality genome [32, 33].

In my job as section editor, but also prior to this, I have encountered many manuscripts in which the authors described only the sequencing and automatic assembly of genomes, often prior to comparative genomics. I have identified many manuscripts that are based on such work, and I have rejected some of them due to lack of basic genome information. Investing a little time in assembly and quality control can resolve assembly mistakes, yielding a lower number of contigs, and can allow identification and closure of plasmids. This little bit of extra time helps editors and reviewers to estimate the quality of genomes used for comparative genomic study, but also the research community to more effectively use genome sequences for various purposes. Problems based on the quality of genome assemblies, as described in this correspondence, would then be minimized. In the end, the benefitfrom good quality genome assemblies in databanks [34, 35] is a win-win situation for all researchers in genomics..

## Data Availability

Not applicable.

## Abbreviations

ANI: Average nucleotide identities
GGDC: Genome-to-genome distance calculation
IS: Insertion sequence

## References

[1] Gurevich A, Saveliev V, Vyahhi N, Tesler G (2013). QUAST: quality assessment tool for genome assemblies. Bioinformatics.

[2] Parks DH, Imelfort M, Skennerton CT, Hugenholtz P, Tyson GW (2015). CheckM: assessing the quality of microbial genomes recovered from isolates, single cells, and metagenomes. Genome Res.

[3] Waterhouse RM, Seppey M, Simão FA, Manni M, Ioannidis P, Klioutchnikov G, Kriventseva EV, Zdobnov EM (2017). BUSCO applications from quality assessments to gene prediction and phylogenomics. Mol Biol Evol.

[4] Mardis ER (2008). Next-generation DNA sequencing methods. Annu Rev Genomics Hum Genet.

[5] Quail MA, Smith M, Coupland P, Otto TD, Harris SR, Connor TR, Bertoni A, Swerdlow HP, Gu Y (2012). A tale of three next generation sequencing platforms: comparison of ion torrent, Pacific biosciences and Illumina MiSeq sequencers. BMC Genomics.

[6] Loman NJ, Misra RV, Dallman TJ, Constantinidou C, Gharbia SE, Wain J, Pallen MJ (2012). Performance comparison of benchtop high-throughput sequencing platforms. Nat Biotechnol.

[7] Rhoads A, Au KF (2015). PacBio sequencing and its applications. Genomics Proteomics Bioinformatics.

[8] Lu H, Giordano F, Ning Z (2016). Oxford Nanopore MinION sequencing and genome assembly. Genomics Proteomics Bioinformatics.

[9] de Lannoy C, de Ridder D, Risse J (2017). The long reads ahead: de novo genome assembly using the MinION. F1000Res.

[10] Gomila M, Busquets A, Mulet M, García-Valdés E, Laculat J (2017). Clarification of taxonomic status within the *Pseudomonas syringae* species group based on a phylogenomic analysis. Front Microbiol.

[11] Konstantinidis KT, Ramette A, Tiedje JM (2006). The bacterial species definition in the genomic era. Phil Trans R Soc B.

[12] Auch AF, von Jan M, Klenk H-P, Göker M (2010). Digital DNA-DNA hybridization for microbial species delineation by means of genome-to-genome sequence comparison. Stand Genomic Sci.

[13] Ruinelli M, Blom J, Smits THM, Pothier JF (2019). Comparative genomics and pathogenicity potential of members of the *Pseudomonas syringae* species complex on *Prunus* sp. BMC Genomics.

[14] Ruinelli M, Blom J, Pothier JF (2017). Complete genome sequence of *Pseudomonas viridiflava* CFBP 1590, isolated from diseased cherry in France. Genome Announc.

[15] Gétaz M, Van der Wolf JM, Blom J, Pothier JF (2017). Complete genome sequences of three isolates of *Xanthomonas fragariae*, the bacterium responsible for angular leaf spots on strawberry plants. Genome Announc.

[16] Darling ACE, Mau B, Blattner FR, Perna NT (2004). Mauve: multiple alignment of conserved genomic sequence with rearrangements. Genome Res.

[17] Rissman AI, Mau B, Biehl BS, Darling AE, Glasner JD, Perma NT (2009). Reordering contigs of draft genomes using the Mauve aligner. Bioinformatics.

[18] Ismail E, Blom J, Bultreys A, Ivanovic M, Obradovic A, van Doorn J, Bergsma-Vlami M, Maes M, Willems A, Duffy B, Stockwell VO, Smits THM, Puławska J (2014). A novel plasmid pEA68 of *Erwinia amylovora* and the description of a new family of plasmids. Arch Microbiol.

[19] Flury P, Aellen N, Ruffner B, Péchy-Tarr M, Fataar S, Metla Z, Dominguez-Ferreras A, Bloemberg G, Frey J, Goesmann A, Raaijmakers JM, Duffy B, Höfte M, Blom J, Smits THM, Keel C, Maurhofer M (2016). Insect pathogenicity in plant-beneficial pseudomonads: phylogenetic distribution and comparative genomics. ISME J.

[20] Rutz D, Frasson D, Sievers M, Blom J, Rezzonico F, Pothier JF, Smits THM (2018). High-quality draft genome sequence of *Pseudomonas wadenswilerensis* CCOS 864^T^. Microbiol Res Announc.

[21] Rutz D, Frasson D, Sievers M, Blom J, Rezzonico F, Pothier JF, Smits THM (2019). High-quality draft genome sequence of *Pseudomonas reidholzensis* strain CCOS 865^T^. Microbiol Res Announc.

[22] Smits THM, Guerrero-Prieto VM, Hernández-Escarcega G, Blom J, Goesmann A, Rezzonico F, Duffy B, Stockwell VO (2014). Whole-genome sequencing of *Erwinia amylovora* strains from Mexico detects SNPs in *rpsL* conferring streptomycin resistance and in the *avrRpt2* effector altering host interactions. Genome Announc.

[23] Smits THM, Rezzonico F, Blom J, Goesmann A, Abelli A, Kron Morelli R, Vanneste JL, Duffy B (2015). Draft genome of the commercial biocontrol strain *Pantoea agglomerans* P10c. Genome Announc.

[24] Palmer M, Steenkamp ET, Coetzee MPA, Blom J, Venter JC (2018). Genome-based characterization of biological processes that differentiate closely related bacteria. Front Microbiol.

[25] Alnajar S, Gupta RS (2017). Phylogenomics and comparative genomic studies delineate six main clades within the family *Enterobacteriaceae* and support the reclassification of several polyphyletic members of the family. Infect Genet Evol.

[26] Tetz G, Vecherkovskaya M, Zappile P, Dolgalev I, Tsirigos A, Heguy A, Tetz V (2017). Complete genome sequence of *Kluyvera intestini* sp. nov., isolated from the stomach of a patient with gastric cancer. Genome Announc.

[27] Pillonetto M, Arend L, Faoro H, D'Espindula HRS, Blom J, Smits THM, Mira MT, Rezzonico F (2018). Emended description of the genus *Phytobacter*, its type species *Phytobacter diazotrophicus* (Zhang 2008) and description of *Phytobacter ursingii* sp. nov. Int J Syst Evol Microbiol.

[28] Blom J, Albaum SP, Doppmeier D, Pühler A, Vorhölter F-J, Zakrzewski M, Goesmann A (2009). EDGAR: a software framework for the comparative analysis of prokaryotic genomes. BMC Bioinformatics.

[29] Blom J, Kreis J, Spänig S, Juhre T, Bertelli C, Ernst C, Goesmann A (2016). EDGAR 2.0: an enhanced software platform for comparative gene content analyses. Nucleic Acids Res.

[30] Seemann T (2014). Prokka: rapid prokaryotic genome annotation. Bioinformatics.

[31] Tetz G, Tetz V (2016). Draft genome sequence of *Kluyvera intestini* strain GT-16 isolated from the stomach of a patient with gastric cancer. Genome Announc.

[32] Phillippy AM (2017). New advances in seuqence assembly. Genome Biol.

[33] Koren S, Harhay GP, Smith TPL, Bono JL, Harhay DM, Mcvey SD, Radune D, Bergman NH, Phillippy AM (2013). Reducing assembly complexity of microbial genomes with single-molecule sequencing. Genome Biol.

[34] Bidartondo MI (2008). Preserving accurary in GenBank. Science.

[35] Harris DJ (2003). Can you bank on GenBank?. Trends Ecol Evol.

